# Functional Characterization of Resistance to Powdery Mildew of *VvTIFY9* from *Vitis vinifera*

**DOI:** 10.3390/ijms20174286

**Published:** 2019-09-01

**Authors:** Yihe Yu, Yutong Wan, Zeling Jiao, Lu Bian, Keke Yu, Guohai Zhang, Dalong Guo

**Affiliations:** 1 College of Forestry, Henan University of Science and Technology, Luoyang 471023, China; 2 Henan Engineering Technology Research Center of Quality Regulation and Controlling of Horticultural Plants, Luoyang 471023, China

**Keywords:** overexpression, powdery mildew, transcription factor, VvTIFY9, *vitis vinifera*

## Abstract

Powdery mildew is a disease caused by fungal pathogens that harms grape leaves and fruits. The *TIFY* gene family is a plant-specific super-family involved in the process of plants’ development and their biotic and abiotic stress responses. This study aimed to learn the function of the *VvTIFY9* gene to investigate molecular mechanisms of grape resistance to powdery mildew. A VvTIFY9 protein encoding a conserved motif (TIF[F/Y]XG) was characterized in grape (*Vitis vinifera*). Sequence analysis confirmed that *VvTIFY9* contained this conserved motif (TIF[F/Y]XG). Quantitative PCR analysis of *VvTIFY9* in various grape tissues demonstrated that the expression of *VvTIFY9* was higher in grape leaves. *VvTIFY9* was induced by salicylic acid (SA) and methyl jasmonate (MeJA) and it also quickly responded to infection with *Erysiphe necator* in grape. Analysis of the subcellular localization and transcriptional activation activity of *VvTIFY9* showed that VvTIFY9 located to the nucleus and had transcriptional activity. *Arabidopsis* that overexpressed *VvTIFY9* were more resistant to *Golovinomyces cichoracearum*, and quantitative PCR revealed that two defense-related genes, *AtPR1* and *AtPDF1.2*, were up-regulated in the overexpressing lines. These results indicate that *VvTIFY9* is intimately involved in SA-mediated resistance to grape powdery mildew. This study provides the basis for exploring the molecular mechanism of grape resistance to disease resistance and candidate genes for transgenic disease resistance breeding of grape plants.

## 1. Introduction

Grape (*Vitis vinifera* L.) is one of the oldest fruit vines in the world. Native to western Asia, it is now cultivated all over the world, where many fungal pathogens can attack it, causing such diseases as downy mildew (*Plasmopara viticola*), powdery mildew (*Erysiphe necator*), and gray mold (*Botrytis cinerea*). Because powdery mildew mainly damages grapes’ leaves and fruits, farmers incur high cost to prevent this disease in their vineyards. To explore molecular mechanisms of grape resistance to powdery mildew, this study aimed to characterize the functioning of the *VvTIFY9* gene.

Plants have evolved mechanisms at the physiological, biochemical, and molecular levels to regulate their resistance to stress factors. Disease resistance mechanisms of plants reportedly include two key defense mechanisms, one relying on pattern recognition receptors (PRRs) that recognize pathogen-associated molecular patterns (PAMPs), to activate the downstream resistance signaling pathway and induce a defense response that prevents the pathogen from invading the known pattern triggering immunity (PTI) [1]. The other involves plant resistance (R) proteins that target pathogen virulence effector proteins to induce effector-triggered immunity (ETI) [2]. In recent decades, studies on the signaling pathways of plant resistance to different microbial pathogens have been conducted extensively. These have shown that effective plant defense against biotrophic pathogens is mainly due to the activation of defense responses regulated by hormone-dependent pathways. The salicylic acid (SA) pathway and jasmonic acid (JA) pathway are two important signaling pathways in gene mediated and induced resistance [3,4]. It is generally believed that SA functions as a major defense hormone against biotrophic and hemibiotrophic pathogens [5], while JA is usually linked to plant resistance against dead nutritional pathogens [6,7].

The TIFY protein family has a conserved TIFY domain, featuring a highly conserved amino acid pattern of TIF[F/Y]XG in its protein sequences and is a plant-specific transcription factor involved in the development of plants and their responses to biotic and abiotic stresses [8,9]. The *TIFY* gene family encodes four subfamilies of proteins: ZML, TIFY, PPD, and JAZ proteins. Many reports have identified and analyzed this gene family in multiple plant species. For example, 28 TIFY family genes were identified in the *Gossypium raimondii* genome and classified into JAZ (15 genes), ZML (8), PPD (3), and TIFY (2). In another work, the expression patterns of TIFY family genes were characterized, revealing that most TIFY family genes were involved in fiber development [10]. For wheat transgenic lines that over-expressed *TdTIFY11a*, they showed higher germination and growth rates under high salinity conditions than wild-type plants did, demonstrating *TdTIFY11a*’s role in wheat’s defense mechanism against salt stress [8]. Similar results have been achieved in *Arabidopsis*, where *AtTIFY10a* and *AtTIFY10b* knockout mutants showed the lower germination rates of under alkaline stress compared to wild type plants. These results provided direct evidence supporting the positive regulatory roles of the TIFY10 proteins in plant responses to alkaline stress [11].

The TIFY gene family has been proven to play a role in the resistance of various plant varieties to biological stress, but most of these studies were carried out on crops or vegetables, with few conducted in cultivated fruit trees or vines [12]. A total of two *TIFY*, four *ZML*, two *PPD*, and 11 *JAZ* genes have been identified in the *Vitis vinifera* genome [13], but the functions of grapevine TIFY transcription factors (TFs) involved in defense response against enemies remain largely unknown. In this study, we found that *VvTIFY9* played an active role in SA-mediated basic defense against grape powdery mildew. The results provide a basis for exploring the molecular mechanisms of disease resistance in grape cultivars and for providing candidate genes to develop plant transgenic disease resistance.

## 2. Results

### 2.1. Characterization of VvTIFY9

**Figure 1:**
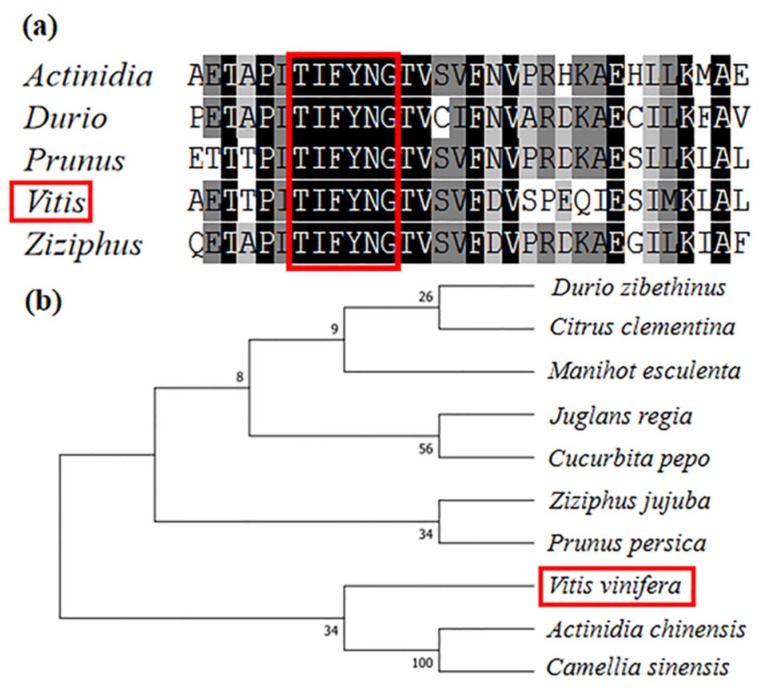
Sequence analysis of VvTIFY9. (**a**) Sequence alignment of the core TIF[F/Y]XG motif conserved motifs from different plant species. The core TIF[F/Y]XG motif (Right) of VvTIFY9 are shown in red frame. (**b**) The phylogenetic relationship of TIFY9 and its closely related homologs from *Vitis vinifera* (Gene ID XP_002262750.1), *Citrus clementina* (Gene ID XP_006438845.1), *Ziziphus jujuba* (Gene ID XP_015902294.1), *Durio zibethinus* (Gene ID XP_022727087.1), *Actinidia chinensis* (Gene ID PSS12092.1), *Prunus persica* (Gene ID XP_007223954.2), *Juglans regia* (Gene ID XP_018859193.1), *Manihot esculenta* (Gene ID XP_021619798.1), *Cucurbita pepo* (Gene ID XP_023547598.1), and *Camellia sinensis.* (Gene ID XP_028072909.1).

*VvTIFY9* encoded a protein of 212 amino acids with a calculated molecular mass of 23.75 kDa and an isoelectric point of 9.24. Nucleotide sequences of *VvTIFY9* identified in *V. vinifera* were compared to others in a phylogenetic tree constructed using TIFY protein sequences of different plant species obtained from a BLAST search in the NCBI database (https//:www.ncbi.nlm.nih.gov). As Figure 1b shows, this phylogenetic analysis showed that the TIFY proteins could be classified into two subfamilies. Alignment with other TIFY protein sequences revealed that VvTIFY9 contains a core TIF[F/Y]XG motif conserved motifs (Figure 1a).

### 2.2. Expression Analysis of VvTIFY9 in Grape

**Figure 2:**
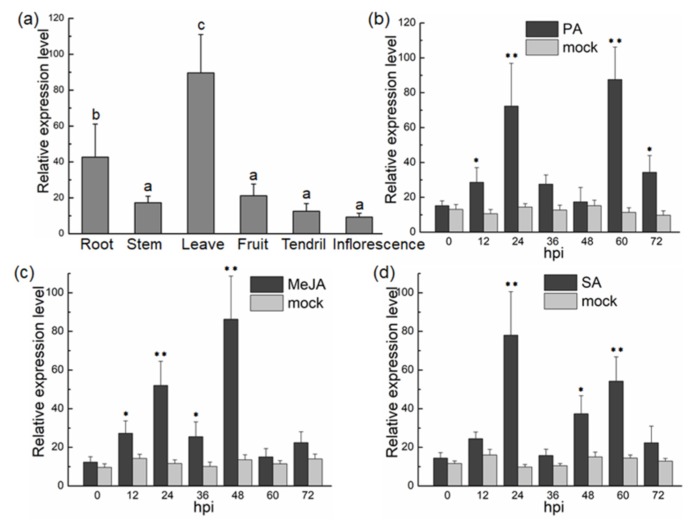
The qRT-PCR analysis of *VvTIFY9* gene expression in grape (*Vitis vinifera* L. cv. Jingxiu). (**a**) The relative expression of *VvTIFY9* in grape root, stem, leaf, fruit, tendril, and inflorescence tissue types, respectively. (**b**) *VvTIFY9* expression in pathogen (PA)-treated plants and normal plants; Jingxiu leaves were infected with *Erisiphe necator* and control leaves (mock) were sprayed with sterile water. (**c**) *VvTIFY9* expression in methyl jasmonate (MeJA)-treated plants and normal plants; leaves were treated with 100 mM of MeJA. (**d**) *VvTIFY9 e*xpression in salicylic acid (SA)-treated plants and normal plants; leaves were treated with 10 mM SA. The control leaves (mock) in (**c**) and (**d**) were sprayed with 0.05% (v/v) *Tween* 20. Expression levels in (**b**), (**c**), and (**d**) were measured every 12 h, for which leaves were collected at different time points as indicated. *VvAct* was as the internal control. Means ± SDs of three independent experiments. Asterisks indicate statistically significant differences vis-à-vis the corresponding control. (* *p* < 0.05, ** *p* < 0.01; Student’s *t*-test).

The quantitative analysis of *VvTIFY9* expression in each tissue type of grape revealed that *VvTIFY9* was more expressed in grape leaves (Figure 2a). To determine whether pathogen infection induces *VvTIFY9* expression, we measured the abundance of *VvTI*FY9 transcripts in Jingxiu leaves at different time points after inoculation with the pathogen (PA) (Figure 2b). These results showed the expression of *VvTIFY9* increased at 12 h post-infection, rising further to 24 h, and peaking at 60 h. To test whether SA or methyl jasmonate (MeJA) induced the gene’s expression, real time qPCR was used to measure *VvTIFY9* transcription in Jingxiu leaves treated separately with these hormones. This showed that *VvTIFY9* expression was induced by MeJA at 12 h, and continued to increase through 24 h, with the highest value reached at 48 h (Figure 2c). Likewise, after the SA treatment, expression of *VvTIFY9* underwent increases in the interval between 12 and 72 h, with two peaks at 24 h and 60 h (Figure 2d). These results indicated that *VvTIFY9* was involved in plant defense responses and responded to multiple defense-related signals, suggesting that *VvTIFY9* could played a role in inducing the defense responses of grapevine.

### 2.3. Subcellular Localization of VvTIFY9

**Figure 3:**
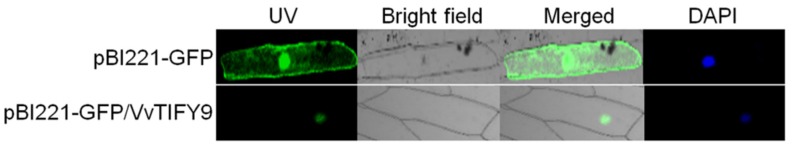
Subcellular localization of VvTIFY9. At 16 h since the transformation, the VvTIFY9-GFP fusion proteins were detected by confocal laser-scanning microscopy. The first row is green fluorescent protein (GFP) alone, and the second row is VvTIFY9 fused with GFP at the C-terminal. The GFP signal (UV), bright field, and a merged image are displayed. The nucleus was stained with DAPI. Data are representative of three independent experiments.

To investigate the subcellular localization of VvTIFY9, a fused gene (pBI221-GFP/VvTIFY9) was transiently introduced into the onion epidermal cells. Expression of GFP alone showed a signal in the nucleus of the onion epidermal cells. Similarly, the GFP signal from epidermal cells expressing pBI221-GFP/VvTIFY9 was also localized to the nucleus (Figure 3).

### 2.4. Transcriptional Activation Analysis of VvTIFY9 in Yeast

**Figure 4:**
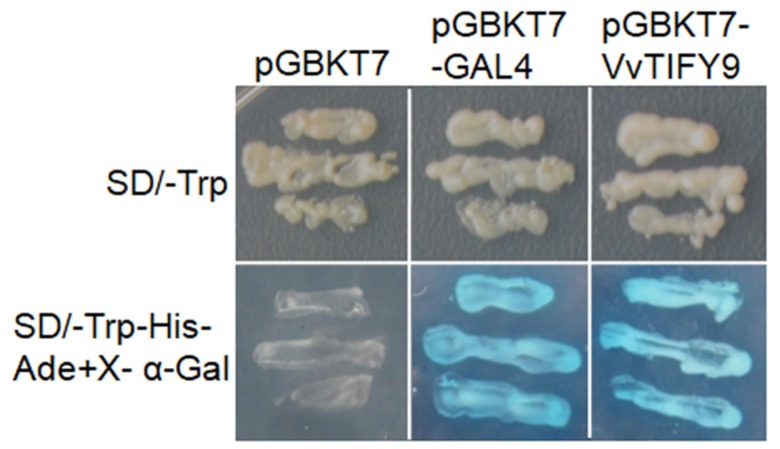
Transcriptional activation analysis of VvTIFY9 in yeast. The full coding sequence of VvTIFY9 was fused into pGBKT7 in frame to generate the pGBKT7-VvTIFY9 structure. Then pGBKT7-GAL4 encoding full-length GAL4 and empty vector pGBKT7 were used as the positive and negative controls, respectively. Yeast were grown on a nonselective medium (SD/-Trp) and selective medium (SD/-Trp-His-Ade+X-α-Gal) plates, incubated at 30 °C for three days before their examination.

To determine whether *VvTIFY9* functions as a transcription factor, a transcriptional activation assay was performed in yeast. This indicated that the full-length VvTIFY9 protein and the positive control (pGBKT7-GAL4) were able to activate the transcription of reporter genes, while the negative control (pGBKT7 alone) showed no such activity (Figure 4).

### 2.5. Overexpression of VvTIFY9 in Arabidopsis Enhanced Its Resistance to A Pathogen

**Figure 5:**
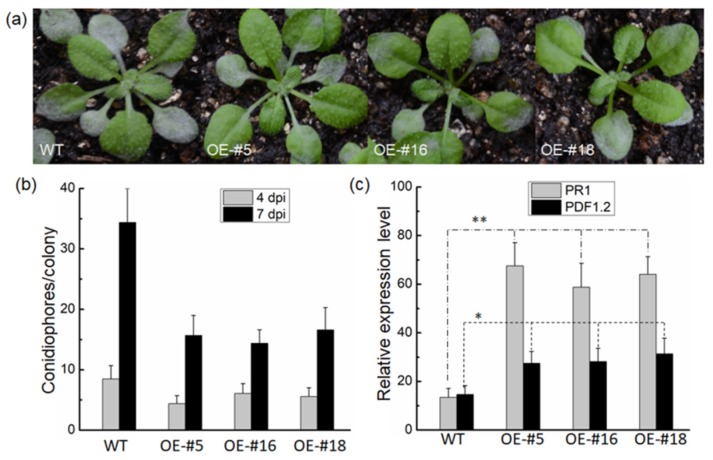
Overexpression of *VvTIFY9* enhanced resistance to powdery mildew in transgenic *Arabidopsis.* (**a**) Phenotype of six-week-old wild-type (WT) and 35S:VvTIFY9 transgenic *Arabidopsis* plants (OE-#5, #16, #18) inoculated with invasive powdery mildew for 14 days. (**b**) Conidiophores content of wild-type (WT) and VvTIFY9 transgenic *Arabidopsis* plants (OE-#5, #16, #18) at 4 and 7 days after inoculation. (**c**) Relative expression levels of PR1 and PDF1.2 in wild-type plants and the overexpressing lines. Means ± SDs from three biological replicates. (* *p* < 0.05, ** *p* < 0.01; Student’s *t*-test).

To distinguish and confirm the specific resistance function of *VvTIFY9* against powdery mildew disease, an overexpression construct of *VvTIFY9* driven by the CaMV-35S was transformed into *Arabidopsis* plants. Three transgenic *Arabidopsis* lines (OE-#5, OE-#16, and OE-#18) were obtained to investigate *VvTIFY9′*s role in basal resistance to powdery mildew, with wild-type plants serving as the control. The content of powdery mildew fungus in wild-type *Arabidopsis* was significantly higher than that in the overexpressing lines (Figure 5a). Further, quantitative analysis of powdery mildew fungus in these wild-type plants and overexpressing lines showed that the latter’s content of conidiophores was significantly lower than the former’s (Figure 5b). *PDF1.2* and *PR1* are key signal molecules in JA and SA signal transduction pathways, respectively [14,15]. Comparing the relative expression levels of *PR1* and *PDF1.2* between wild-type plants and overexpressing lines revealed the latter had significantly higher *PR1* expression (Figure 5c).

### 2.6. Overexpression of VvTIFY9 Increased the SA Content in Arabidopsis

**Figure 6:**
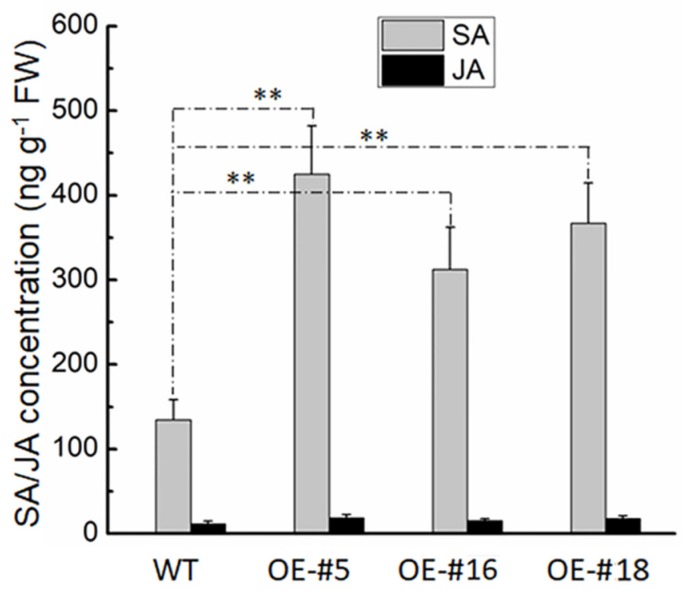
Endogenous levels of free salicylic acid (SA) and jasmonate (JA). Free SA and JA in *Arabidopsis* wild-type (WT) and overexpressing (OE-#5, #16, #18) plants were quantitatively analyzed. Means ± SDs from three biological replicates. (** *p* < 0.01; Student’s *t*-test).

To test whether VvTIFY9 is involved in the biosynthesis of plant endogenous hormones, we determined the SA and JA concentration in *VvTIFY9* transgenic plants and the wild type. As Figure 6 shows, the SA content of overexpressing plants was significantly higher than that of wild-type plants, whereas no significant difference was found for their JA content.

## 3. Discussion

The TIFYs constitute a plant-specific super-family of proteins, which are closely involved in plant development and responses to biotic and abiotic stresses. TIFY super-family encodes four subfamilies, including ZML, JAZ, PPD, and TIFY [16]. For a number of plant species, studies have reported members of this super-family but the focus has been on the JAZ and PPD subfamilies leaving the TIFY subfamily less investigated. In our study *VvTIFY9* was cloned from grapevine and its function in this plant’s defense response identified. Comparing expression of this gene among tissue types indicated that *VvTIFY9* is mainly expressed in the leaves (Figure 2). In rice, 20 *OsTIFY* genes were identified, of which seven (2a, 2b, 3, 6a, 6b, 10a, and 11b) were found to express at high levels in leaves [17]. In maize, 22 genes were detected in most of its tissues, yet the transcription levels of *ZmTIFY15* and *ZmTIFY25* were higher in seedlings, leaves, and other vegetative organs [18]. Gene expression profiling indicated that *VvTIFY9* was affected by the defense signaling molecules SA and MeJA (Figure 2). Previous studies have shown that the TIFY family gene is the key factor in the jasmonate signaling pathway [19,20]. In addition, the *TIFY* gene of grape is regulated by JA and ABA (abscisic acid), but not by SA [13]. It is well known that grape powdery mildew mostly occurs in grape leaves; this is precisely where *VvTIFY9* was most highly expressed in leaves, inducible by both SA and the pathogen. Hence, we reasonably speculated that *VvTIFY9* could be involved in grape’s defense response.

Studies have shown that transcription factors are crucially involved in various activities of plants, including their stress responses. Most transcription factors are located in the nucleus. For example, OsWRKY77 is located in the nucleus of onion epidermal cells and is capable of transcriptional activation [21]. Similarly, VvZFP11 is only located in the onion epidermal nucleus but functions there to repress transcription. [22]. To identify whether VvTIFY9 functions as a transcription factor, we carefully examined its subcellular localization and analyzed its transcriptional activation activity, finding it localized in the nucleus where it displayed activation activity in yeast (Figure 3; Figure 4). These results confirm that VvTIFY9 may indeed function as transcription factor. Several other studies have identified the participation of TIFY transcription factors in plants’ abiotic stress and development [10,11], but prior to our study the functioning of these in grapevine for its defense response was largely unknown.

Many studies verify a gene’s function by observing the growth, development, and stress tolerance phenotype in overexpressing plants. In rice, overexpression of *OsTIFY11b* increased its grain size and weight through an enhanced accumulation of transient carbohydrate reserves and culm [23], and overexpression of *OsTIFY11a* markedly increased its tolerance of salt and dehydration stress [17]. We found that infection with powdery mildew (*Erysiphe necator*) significantly induced gene expression of *VvTIFY9* (Figure 2) and its overexpression in *Arabidopsis* enhanced resistance to powdery mildew while promoting the expression of two defense-related marker genes (*PR1* and *PDF1.2*). Furthermore, the expression levels of *PR1* exceeded those of *PDF1.2* (Figure 5), and the SA content was higher in overexpressed than wild-type plants, which had similar JA contents (Figure 6). Plant hormones are viral for regulating developmental processes and signaling networks involved in plant responses to a wide range of biotic and abiotic stresses [24]. In *Arabidopsis*, JAZ10 is a negative regulator of both JA signaling and disease symptom development [25]. In grape, several TIFY genes—mainly JAZ genes—were highly responsive to certain types of abiotic stress and hormone treatments, and likewise to JA and ABA but neither SA nor ET [13]. A. Cuéllar Pérez [26] demonstrated the TIFY8 gene was critical for nuclear signal transduction, yet JA had no effect on TIFY8 at either the transcription or post-transcription level. Based on the comprehensive analysis of experimental data in our study, *VvTIFY9* of grapes clearly participated in SA mediated powdery mildew resistance as a transcription factor. However, the specific molecular mechanism by which *VvTIFY9* strengthens the grapevine’s resistance to powdery mildew remains to be elucidated in further research.

## 4. Materials and Methods

### 4.1. Plant Material and Growth Conditions

*Vitis vinifera* L. cv. Jingxiu were grown in a soil mixture (perlite: vermiculite: loam soil, 1:1:3, v/v/v) in a culture room (25 °C; photoperiod of 14 h/10 h; light intensity of 100 mol m^−2^ s^−1^) [27]. Wild-type (WT) and overexpression (OE-#5; 16; 18) *Arabidopsis* plants were grown in a climate chamber (22 °C; photoperiod of 16 h/8 h; light intensity of 130 μmol m^−2^ s^−1^; 65%). *Vitis vinifera* L. cv. Cabernet Sauvignon vines were grown in natural conditions.

### 4.2. Pathogen Inoculation and Exogenous Hormone Treatment

Field samples of *E. necator* were collected from twelve years old Cabernet Sauvignon plants growing at the Zhoushan campus of Henan University of Science and Technology, in Luoyang city of Henan Province. The *E. necator* challenge infection of Jingxiu leaves was conducted as previously described [28]. A total of 60 grapevine vines were used in this study, of which 15 were treated with powdery mildew, 15 were treated with SA, 15 were treated with MeJA, and 15 were used in the control group. Either 10 mM SA or 100 mM MeJA solutions with *Tween* 20 (0.05%, *v*/*v*) were sprayed onto grapevine leaves of the same age. *Tween* 20 (0.05%, *v*/*v*) alone was sprayed on grape leaves to serve as the control. Grape leaves were collected at 0, 12, 24, 36, 48, 60, and 72 h post-inoculation (hpi) and immediately frozen in liquid nitrogen. *Golovinomyces cichoracearum* (UCSC1 isolate) was cultivated on *Arabidopsis phytoalexin deficient 4* (*pad4*) mutant plants.

### 4.3. Real-Time Quantitative PCR Assays

Total RNA samples of *Vitis vinifera* L. cv. Jingxiu leaves and fruits were extracted with a RNAprep Pure Plant Kit (Tiangen, Beijing, China), from which first-strand cDNA was synthesized using a PrimeScript 1st strand cDNA synthesis kit (TaKaRa, Dalian, China). The full-length cDNA of *VvTIFY9* was then amplified using gene-specific primers (Table S1) and real-time quantitative PCR (qPCR) performed in a Bio-Rad IQ5 real-time PCR detection system (Bio-Rad Laboratories, Hercules, CA, USA). PCR amplifications used high-fidelity thermostable DNA polymerase (Takara, Dalian, China), in a total system volume of 50 µL. PCR reaction conditions were 94 °C for 5 min, 94 °C for 30 s, 58 °C for 30 s, and 72 °C for 2 min in 30 cycles, followed by 5 min at 72 °C [29]. The relative expression of the target gene was calculated using the 2^−ΔΔCt^ method [30]. All reactions were performed in triplicate.

### 4.4. Sequence Comparison and Phylogenetic Analysis

Full amino acid sequences of each TIFY member were aligned by the ClustalX program [31,32]. For these, a neighbor-joining phylogenetic tree was constructed in MEGA 7 software with a bootstrap test (*n* = 1000 times), pair wise deletion, and a Poisson model [33].

### 4.5. Subcellular Localization

The coding sequence of *VvTIFY9* without its termination codon was cloned into the *Xba* I/*Kpn* I sites of the pBI221-GFP vector, thus generating the pBI221-GFP/VvTIFY9 fusion with the CaMV 35S promoter. Specific primers containing the *Xba* I and *Kpn* I sites are listed in Table S1. The recombinant vector was verified by sequencing it three clones. The fusion construct was transformed into onion epidermal cells via particle bombardment, by using a *Bio-Rad* biolistic *PDS 1000/He* system (PDS-1000, Bio-Rad). The pBI221-GFP vector served as a control. Transformed materials were incubated in darkness in a growth chamber (24 °C, 16–18 h), and the nuclear DNA stained with 4′, 6-diamidino-2-phenylindole (DAPI). A *Zeiss LSM* 510 confocal laser microscope (Zeiss, Oberkochen, Germany) was used to detect green fluorescent protein (GFP) signals. The resulting plot is representative of three similar individually acquired images.

### 4.6. Transcription Activation Assay in Yeast

The VvTIFY9 ORFs were cloned into the *Nde*I/*BamH*I sites of the pGBKT7 vector, to create the pGBKT7-VvTIFY9 constructs. Producing pGBKT7-GAL4 as the positive control, the full-length GAL4 sequence from pCL-1 was cloned into pGBKT7; the pGBKT7 empty vector served as the negative control. All above constructs were transformed into the yeast strain AH109 and grown on SD/-Trp or SD/-Trp-His-Ade+X-α-Gal plates. Primer sequences used are listed in Table S1.

### 4.7. Plant Expression Vector Construction and Arabidopsis Transformation

The full-length cDNA of *VvTIFY9* was amplified from *V. vinifera* cv. Jingxiu by reverse transcription polymerase chain reaction (RT-PCR). The ensuing PCR fragment was confirmed by sequencing and then directionally cloned into the pCAMBIA2301 vector, to create the pCAMBIA2301-VvTIFY9 construct. This constructed plasmid was introduced into *A. tumefaciens* GV3101 cells by electroporation. Transformation of *Arabidopsis* was performed by the floral dipping method [34]. Positive transgenic lines were first screened on Kana plates, then identified by RT-PCR, and T3 homozygous transgenic lines selected to assess disease resistance.

### 4.8. Quantification of Endogenous JA and SA.

*Arabidopsis* leaves were weighed at 300 mg and then frozen and ground in liquid nitrogen. The resulting powder was evenly mixed with 4 mL of high performance liquid chromatography (HPLC) grade methanol (Sigma-Aldrich, Shanghai, China) and kept at 220 uC for 12 h. [35,36]-dihydro-JA (300 ng) and d6-SA (500 ng) were each added to the ground mixture as as internal standards. The mixture was shaken in darkness at 4 °C for 12 h and centrifuged at 18,000 g for 10 min at 4 °C. Supernatant was collected, and 1 mL ethyl acetate (sigma-aldrich) was used for secondary extraction of the precipitation, shaken for 2 h, followed by centrifugation at 4 °C for 10 min. The two supernatants were mixed and evaporated to a to dryness under exposure to N 2 gas. Then the residue was re-suspended in 0.5 mL of 70% methanol and centrifuged at 4 °C for 2 min at 18,000 g. Samples were analyzed in a GC/MS system (6890 N/5973 MSD, Agilent Technologies, Inc., Palo Alto, CA, USA) equipped with an HP-5-MS column (30 m by 0.25 mm by 0.25 mm; 19091S-433, J&W Scientific, Agilent Technologies). Endogenous JA, SA, and their internal standards were each analyzed in the full-scan mode. All experiment has three sample replicates for the analyses [37,38].

## 5. Conclusions

In this study we analyzed the functional relevance of the *VvTIFY9* gene for conferring plant disease resistance. Expression in different grape tissues indicated that *VvTIFY9* was mainly expressed in leaves, which is also where grape powdery mildew mainly occurs. Gene expression profiling indicated that *VvTIFY9* activity is affected by the defense signaling molecules SA and MeJA. Subcellular localization and transcriptional activation results showed that *VvTIFY9* functions as a transcription factor. Overexpressing *VvTIFY9* in *Arabidopsis* proved *VvTIFY9′*s involvement in SA-mediated resistance to grape powdery mildew in grape plants. Our study provides a basis for exploring the molecular mechanism of grape’s resistance to fungal disease and candidate genes for breeding transgenic disease resistance in grape cultivars. The molecular mechanism of transcription factors regulating downstream target genes will be further elucidated.

## Supplementary Materials

Supplementary materials can be found at https://www.mdpi.com/1422-0067/20/17/4286/s1. Table S1. The sequences of the primers used in these experiments.

## Abbreviations

SA	salicylic acid
MeJA	methyl jasmonate
PA	*Erysiphe necator*
PRRs	pattern recognition receptor
PAMPs	pathogen-associated molecular pattern
PTI	pattern triggering immunity
ETI	effector-triggered immunity
JA	jasmonic acid
TFs	transcription factors
*pad4*	*phytoalexin deficient 4*
DAPI	4′, 6-diamidino-2-phenylindole
GFP	green fluorescent protein
RT-PCR	reverse transcription polymerase chain reaction
