# Supplementary material for: Functional Characterization of Resistance to Powdery Mildew of VvTIFY9 from Vitis vinifera
Source: Int J Mol Sci. 2019 Sep 1;20(17):4286. doi: 10.3390/ijms20174286 (PMC6747219; doi:10.3390/ijms20174286)
Supplement: Supplementary file 1 [file ijms-20-04286-s001.pdf]

Table S1. The sequences of the primers used in these experiments

Primer name	oligonucleotide primers	Purpose or vector
VvTIFY9-F	ATGTCGAGAGCCGCCATGGAA	PCR
VvTIFY9-R	CTAGGCCCGGAACAGCTCCT	
VvTIFY9qt-F	ATAATCCGCCGTTCGCTT	qtRT-PCR
VvTIFY9qt-R	CACAGTCTTGGTGCTTCCGT	
VvUbi-F	GTGGTATTATTGAGCCATCCTT	qtRT-PCR
VvUbi-R	AACCTCCAATCCAGTCATCTAC	
VvTIFY9-XbaI-F	GGCTCTAGA ATGTCGAGAGCCGCCATGGAA	pBI221-GFP
VvTIFY9-KpnI-R	GCGGGTACCGGCCCGGAACAGCTCCT	
VvTIFY9-NdeI-F	GGGCATATGATGTCGAGAGCCGCCATGGAA	pGBKT7
VvTIFY9-BamHI-R	GGGGGATCCGGCCCGGAACAGCTCCT	
AtPR1-F	GGAGCTACGCAGAACAACTAAGA	qRT-PCR
AtPR1-R	CCCACGAGGATCATAGTTGCAACTGA	
AtPDF1.2-F	TCATGGCTAAGTTTGCTTCC	qRT-PCR
AtPDF1.2-R	AATACACACGATTTAGCACC	
β-TUB 4-F	GAGGGAGCCATTGACAACATCTT	qRT-PCR
β-TUB 4-R	GCGAACAGTTCACAGCTATGTTCA	
